# Supplementary material for: Subthalamic, not striatal, activity correlates with basal ganglia downstream activity in normal and parkinsonian monkeys
Source: eLife. 2016 Aug 23;5:e16443. doi: 10.7554/eLife.16443 (PMC5030093; doi:10.7554/eLife.16443)
Supplement: Figure 12—source code 1. — DOI: http://dx.doi.org/10.7554/eLife.16443.021 [file elife-16443-fig12-code1.docx]

%%%%%%%%%%%%%%%%%%%%%%%%%%%%%%Custom_made_artefact_removal_procedure%%%%%%%%%%%%%%%%%%%%%

clear all

close all

% Load figure 12 - Source data 1.xlsx

[NUMERIC,TXT,RAW]= xlsread('Figure 12 - Source data 1.xlsx');

% Set from NUMERIC variable the first and last raws and the first and last columns to define the matrix corresponding to all the power spectrum densities (PSDs) of one neuronal population (MSNs, TANs, STN, GPe or SNr neurons) in a specific state (before or after MPTP).

% See below, the example for PSDs of the LFPs recorded in the vicinity of the SNr neurons after MPTP intoxication.

raw_first = 434;

raw_last = 554;

column_first = 220;

column_last = 436;

data = NUMERIC (raw_first:raw_last, column_first:column_last);

Dim_data = size(data);

peak_database = zeros (Dim_data(1),Dim_data(2));

for i = 1:Dim_data(1)

for j = 1:Dim_data(2);

if j == 1

peak_database(i,j) = 0;

elseif j == 2

peak_database(i,j) = 0;

elseif j == 216

peak_database(i,j) = 0;

elseif j == 217

peak_database(i,j) = 0;

elseif data(i,j)> data(i,j-1) && data(i,j)> data(i,j+1);

peak_database(i,j) = 1;

end

PeakIndex{i} = find(peak_database(i,:)== 1);

end

for k = 1:length(PeakIndex{1,i})

l{k} = find(data(i,PeakIndex{1,i}(k))> 2*((data(i,PeakIndex{1,i}(k)-2)+ data(i,PeakIndex{1,i}(k)+2))/2));

Artefact_Index = find (cellfun (@isempty,l)== 0);

real_Artefact_Index = PeakIndex{1,i}(Artefact_Index);

end

remove_artefact_idx = [real_Artefact_Index - 2,real_Artefact_Index - 1,real_Artefact_Index,real_Artefact_Index + 1,real_Artefact_Index + 2];

remove_artefact_idx = sort (remove_artefact_idx);

data(i,remove_artefact_idx)= NaN;

clear ('l','Artefact_Index','real_Artefact_Index', 'remove_artefact_idx')

% Figure

figure (1)

hFig = figure(1);

set(gcf,'PaperPositionMode','auto')

set(hFig, 'Position', [680 558 560 420])

freq_axis = linspace (3,75,217);

plot(freq_axis,data(i,:))

ylim([0 0.12])

xlim ([3 75])

xlabel('frequency (Hz)')

set(gca,'xscale','log')

ylabel('power')

hold on

set(gca,'FontSize',12,'LineWidth',1,'box','off')

end
